# Surgery

**Published:** 1912-12

**Authors:** C. Ferrier Walters


### SURGERY.

**The Surgery of the Vascular System,** judging from the scarcity of records, has not made the same advance in this country as in those in which the development of its technique on animals is not hampered by so many legal restrictions.

Most of these notes on work on the suture of vessels are abstracted from contributions by Watts[1] and by Ehrenfried and Boothby,[2] to which reference may be made for the complete bibliography of recent work in this subject.

As far back as 1759 a record is found of the successful

[1] *Johns Hopkins Hosp. Bull.*, 1907, xviii. 153.
[2] *Ann. Surg.*, 1911, liv. 485.

closure of the brachial artery by Hallowell, who, at Lambert's suggestion, inserted a steel pin three-eighths of an inch in length through the edges of a longitudinal wound of that vessel, and round this, after the manner in which a hare-lip pin is used, wrapped a figure-of-eight suture. Nearly a hundred years later Wattmann applied with success lateral ligatures to wounded veins, and Eck, in 1877, experimenting on dogs for a way to relieve portal congestion clinically, on numerous occasions, anastomosed the vena cava and portal vein by a method almost identical with that used at the present day for lateral intestinal anastomosis, save that all sutures were placed before tying, and the vessels were divided at the last moment by a special form of scissors.

Ten years after these advances in the surgery of the blood-vessels, von Horoch made an unsuccessful attempt at end-to-end suture of an artery, owing to interference with its patency. The first successful end-to-end anastomosis of an artery, with fine silk, over a glass bobbin, was obtained in 1894 by Abbé of New York.

Queirolo and Masini, in 1895, elaborated a method in which the vessel was pushed through a glass tube of slightly larger calibre, its end cuffed back over this, and the other end of the divided vessel applied over this cuff and there tied.

Payr, four years after these experiments, and apparently without knowledge of their methods, used absorbable magnesium protheses in the same manner.

Murphy,[1] shortly after, experimented with considerable success by means of invagination and direct suture without any mechanical aid; and Jaboulay and Briau developed experimentally, anastomosis by interrupted suture, invaginating the intima, and by this means obtained specimens with intima intact at the site of union, and without thrombosis.

Jassinowsky placed simple suture on a sound basis, but it is to Carrel,[2] who in 1902 improved the methods of Jaboulay and Briau, that is due the modern method of circular suture, without a complete knowledge of which failure almost always follows attempts at end-to-end union.

In laceration of arterial trunks, where a considerable area of vessel wall is damaged, it has been found possible to excise this, and perform an end-to-end anastomosis, especially if the injury chance to be at the flexion point of the limb, and tension can be thus avoided after suture.

Numerous cases have now been reported of the clinical success of Carrel's method of end-to-end suture, and

[1] Murphy, "Resection of Arteries and Veins in Continuity—End-to-end Anastomosis," *Med. Rec.*, 1897, li. 73–88.

[2] Carrel and Guthrie, "Uni and Bituminal Venous Transplantation," *Surg. Gyn. and Obst.*, 1906, ii. 266–86.

satisfactory results have followed his attempt to transplant a piece of vein between the ends of an artery, a portion of which has been removed because of injury to its walls, and where an attempt at direct union would end in inevitable failure, from too much tension.

On Payr's previously-mentioned method of end-to-end arterial anastomosis, Crile[1] has developed direct transfusion. To Payr's protheses, which consist of metal tubes (of a diameter varying from 2 m.m. upwards and three-eighths of an inch in length, according to the size of the vessels to be utilised, and which have two grooves in which the vessels are tied) he added a small angular metal handle to facilitate its introduction.

The reported cases where this method of transfusion has been employed have shown a rapid rise in the blood count and the hæmoglobin content, together with the immediate disappearance of the ischæmic symptoms ; but on the other hand, normal blood transferred in some hæmic diseases appears to act as a hæmolysin, and the recipient's condition becomes worse.

Longitudinal or transverse wounds of arteries may be closed by through and through continuous sutures, after the method of Crile, for the union of completely divided vessels, but Dorrance[2] has devised a method which, while the suture traverses all the coats of the vessel, unlike Crile's it is not in contact with the blood stream. It can be applied to partially or completely divided vessels.

One might here mention that Brewer[3] has recently devised a method of closing longitudinal wounds in arteries by strips of adhesive plaster, made of zinc oxide spread on thin sheets of pure rubber sterilised by formaldehyde vapour. Experimentally the results were very good, but clinically this method was a failure, though it is still possible that with a more reliable material some use may be made of it as a support to sutured vessels, or to veins implanted between divided arteries.

Another direction in which the surgery of the vessels has progressed is in arterio-venous anastomosis, which has been utilised apparently with some success in severe injuries to limbs, with obliteration of the blood supply at the site of injury, and in thrombosis and embolism with threatened gangrene.

The method is by end-to-end suture as previously described of both ends of the vein to both ends of the artery, unless the vessels are of different calibre, when invagination of the smaller vessel into the lumen of the larger, followed by circular suture, is the method found to be most successful.

[1] Crile, "Direct Transfusion," *Ann. Surg.*, 1907, xlvi. 329.

[2] Dorrance, "New Method of Suture of Arteries," *Ann. Surg.*, 1906, xliv. 409.

[3] Brewer, *Ann. Surg.*, 1904, xl. 856.

The circulation is usually re-established in three hours by forcing of the venous valves, and a line of demarcation appears, in a previously spreading gangrene.

Lateral arterio-venous anastomosis is unsatisfactory, as most of the blood tends to go through the proximal end of the vein to the heart and not to the limb, but if the vessels are very different in calibre the smaller may be implanted into the side of the larger vessel.

Arteriotomy for traumatic thrombosis or embolism has been practised, and should find a place in vascular surgery, particularly for the latter condition.

Traumatic thrombosis usually due to rupture of the intima does not so readily lend itself to a favourable issue, as the thrombus tends rapidly to recur owing to the presence of a roughened or partially detached intima, and it is in such a condition that excision of the damaged portion and end-to-end union, or transplantation of a vein, may overcome the difficulty.

The treatment of embolism by arteriotomy appears more likely to be successful, for where we have an undamaged intima thrombosis is less likely to occur; but a difficulty arises as to the localisation of such emboli, for the severe pain present in such a condition apparently indicates rather the site at which the ischæmic process ends than that of the embolus.

Sabanajew, in 1896, failed to find an embolus at the site of the pain, and Stewart,[1] after opening the popliteal artery for a supposed embolus at that site found it in the femoral artery just below Poupart's ligament.

A matter of the greatest importance in the consideration of the amputation of limbs and the ligation of the great vessels for aneurysm, is the *ante operationem* determination of the efficiency or otherwise of the collateral circulation.

In children there is seldom any doubt of this, the elasticity of their circulatory powers being well known; but in adults, especially where senile sclerotic and thrombotic gangrene, aneurysm, or trauma of the vessels exists, a previous knowledge of the collateral circulation is all important in deciding the yes or no and the site of amputation.

With a view to estimating the extent and flexibility of a collateral circulation, Matas[2] has adopted the hyperæmic test of Moskowicz[3] to aneurismal conditions, and to prove the extent of viable tissue in senile arterio-sclerotic and thrombotic gangrene. His results have amply proved the efficiency of his method. The procedure is as follows: he found— after having exsanguinated a healthy limb by raising it and applying an Esmarch's bandage from its extremity to its root,

[1] Stewart, "Arteriotomy," *Ann. Surg.*, 1907, xlvi. 339.

[2] Matas, *Ann. Surg.*, 1911, liii. 1.

[3] Moskowicz, *Mitt a. d. Grenzgeb. d. Med. u. Chir.*, 1907, xvii. 216.

and having obliterated its main artery, at the top of the Esmarch, by applying to it a tourniquet which pressed on that artery only, leaving the rest of the circumference of the limb free, that on removing the Esmarch—the tourniquet remaining *in situ*—the collateral circulation quickly establishes a blood supply to the whole limb in from three to eight minutes, as manifested by a blush spreading to the extremity of the toes or fingers.

This method, applied to a limb with commencing gangrene, demarcates almost accurately the viable from the dead tissues, and indicates the line at which amputation may be safely undertaken without fear of the gangrenous process spreading to the flaps, and prevents the unnecessary sacrifice of parts which must undoubtedly have happened on many occasions in order to get well above the site of a possible spread of the ischæmic process, and the gangrene which follows in its footsteps.

In cases in which the application of this method proves the existence of an inefficient collateral circulation, Matas and Halsted have devised a means of developing such a collateral circulation, based on the work of Kokuri, Sargo and Tanaka, who, during the Russo-Japanese War, before ligaturing great vessels for aneurism, etc., developed the collateral circulation by systematic compression of the main trunk for days before the operation.

Both Matas[1] and Halsted, working independently, have by means of aluminium and silver bands, of the same breadth as the diameter of the vessel, exercised varying degrees of compression on large trunks for as much as three or four days together, without injury to the intima, with a view to developing the collateral circulation, and so avoiding the dire after-results not unfrequently following on ligature of the carotid, or the main vessel of a limb.

One might here also mention Matas's well-known work on the treatment of aneurisms by endo-aneurysmorrhaphy, by which method 149 cases of aneurism were treated, with an occurrence of gangrene in five cases only.

A new method of dealing with varicose veins of the leg, in which Trendelenburg's sign is present, has been recorded by Gene and Hesse and Schaack.[2] The dilated and incompetent saphenous vein is implanted laterally into the femoral vein some four inches below the saphenous opening.

Above this site in the femoral vein, almost constantly, are present from two to four pairs of valves, which explains the treatment.

Up to the present forty-five cases of this end-to-side anastomosis have been reported, but an insufficient time has

[1] Matas, *J. Am. M. Ass.*, 1908, li. 1667.

[2] Hesse and Schaack, *Arch. f. klin. Chir.*, 1911, xcv. 381-428.

elapsed to prove the efficiency or otherwise of this method of dealing with varicose veins of the lower extremity, and, moreover, one death occurred amongst these from septic thrombosis and septicæmia, and to those inexperienced in such anastomoses the likelihood of failure owing to thrombosis appears to be considerable. Time only can prove whether this proceeding is justifiable and its results better than the often but temporary relief only, effected by the usual methods of dealing with this troublesome affection.

As Lane has said with respect to bone plating, it is not the casual aseptic methods of abdominal surgery that avail in the surgery of the vascular system; only a perfectly aseptic technique can succeed.

As a result of the improved methods, and with these, increased success in the surgery of the vessels, has arisen the successful transplantation of organs, homonymous transplantation of the kidney is an accomplished fact, and there is promise in the future of an ability to transplant entire limbs, partial successes in this direction having been already obtained.

C. FERRIER WALTERS.

---

